# Two novel missense variants in *SPTBN2* likely associated with spinocerebellar ataxia type 5

**DOI:** 10.1007/s10072-021-05204-3

**Published:** 2021-04-02

**Authors:** Xianli Bian, Shang Wang, Suqin Jin, Shunliang Xu, Hong Zhang, Dewei Wang, Wei Shang, Ping Wang

**Affiliations:** Department of Neurology, The Second Hospital of Shandong University, Jinan, 250033 Shandong China

**Keywords:** Spinocerebellar ataxia type 5, Spectrin beta nonerythrocytic 2, Cerebellar ataxia, Targeted next-generation sequencing

## Abstract

**Introduction:**

Spinocerebellar ataxias (SCAs) are a heterozygous group of neurodegenerative disorders. Spinocerebellar ataxia type 5 (SCA5) is a rare autosomal-dominant ataxia with pure cerebellum involvement. The clinical characteristics are limb and gait ataxia, trunk ataxia, sensory deficits, abnormal eye movement, dysarthria, and hyperactive tendon reflexes. Spectrin beta nonerythrocytic 2 gene (*SPTBN2*), coding β-III spectrin protein, was identified to be associated with SCA5. To date, more than 19 variants of *SPTBN2* have been reported.

**Methods:**

A family and an apparently sporadic patient with ataxia and cerebellar atrophy were recruited from Shandong Province (China). To discover the disease-causing variants, capillary electrophoresis and targeted next-generation sequencing were performed in the proband of the family and the sporadic patient. The candidate variants were verified by Sanger sequencing and analyzed by bioinformatics software.

**Results:**

In our study, we verified two novel heterozygous variants in *SPTBN2* in a SCA pedigree and a sporadic patient. The proband of the pedigree and her mother presented with walking instability and progressively getting worse. The sporadic patient suffered from slurred speech, walking instability, and drinking water choking cough. MRI examination of the proband and sporadic patient both displayed moderate cerebellar atrophy. The variants identified were traditionally conserved and predicted probably damaging and disease-causing by bioinformatics analysis.

**Conclusion:**

We identified two novel heterozygous variants of *SPTBN2* resulting in severe ataxia which further delineated the correlation between the genotype and phenotype of SCA5, and pathogenesis of variants in *SPTBN2* should be further researched.

## Introduction

Spinocerebellar ataxias (SCAs) are a heterozygous group of neurodegenerative disorders, which are characterized by cerebellar ataxia, dysarthria, and difficulty in swallowing. SCAs have high genetic and clinical heterogeneity, and there are more than 40 subtypes of SCAs so far. More than 20 loci have been confirmed to be involved in the occurrence of SCAs [1]. Spinocerebellar ataxia type 5 (SCA5) is a rare autosomal-dominant ataxia that purely targets the cerebellum [2], and the worldwide prevalence is < 1%. Onset of SCA5 usually occurs in the early 30s, although it ranges from infancy to 68 years [3]. The predominantly clinical manifestations of SCA5 are limb and gait ataxia (> 90%); however, some patients also have trunk ataxia, sensory deficits, abnormal eye movements, dysarthria, and hyperactive deep tendon reflexes (25–90%) [4].

Spectrin beta nonerythrocytic 2 gene (*SPTBN2*), which encodes β-III spectrin, has been known as causative gene for SCA5. β-III spectrin is a 2390-amino acid protein including two calponin-homology (CH) domains at the N-terminal, 17 spectrin repeats, and a pleckstrin-homology (PH) domain at the C-terminal [5]. It is highly expressed in Purkinje cells and stabilizes membrane proteins, including glutamate receptors [6]. Currently, 20 variants in *SPTBN2* were discovered to be associated with SCA5.

In the present study, we described a family and an apparently sporadic patient from Shandong Province (China) with ataxia and cerebellar atrophy. Two heterozygous variants were found using targeted next-generation sequencing.

## Materials and methods

### Research subject

A four-generation family with autosomal-dominant SCA5 and an apparently sporadic patient were contacted in Shandong Province, China. A written informed consent was obtained from all the participants, and the study was approved by the ethics committee of the Second Hospital of Shandong University. The genomic DNA samples were obtained from peripheral blood by standard techniques. All the participants underwent clinical, laboratory, cognitive function, and magnetic resonance imaging (MRI) examinations. The laboratory and MRI examinations were performed in the Second Hospital of Shandong University. Clinical and cognitive function examinations were carried out by an experienced neurologist.

### DNA extraction

The whole-genomic DNA was extracted from peripheral blood of individuals by using the Wizard genomic DNA purification kit (Promega) according to the standard manufacturer’s protocol.

### Targeted next-generation sequencing

Targeted next-generation sequencing was performed in the genomic DNA of the proband and sporadic patient. Target sequences which included 730 known genes for hereditary ataxia were enriched by using customized capture probes chips (Illumina, San Diego, CA). The genomic DNA sample was randomly fragmented. Extracted DNA was processed by ligation-mediated PCR (LM-PCR) amplified, purified, and enriched. Captured LM-PCR products were analyzed using the Agilent 2100 Bioanalyzer and then loaded on Hiseq2000 platform for high-throughput sequencing. Raw data was aligned with the human hg19 reference genome using BWA software (Burrows Wheeler Aligner). GATK software (Genome Analysis Toolkit) was used to analyze the SNVs (single nucleotide variants) and frame-shift variants (insertion and deletion) in the genome. Then the population database 1000 Genomes (1000 human genome dataset), Genome AD (Genome Aggregation Database dataset) 2.1.1, and ExAC (The Exome Aggregation Consortium dataset) were used to filter the analyzed SNVs and in-frame variants. Candidate variants were screened by Human Mendelian Inheritance Database (OMIM), Human Gene Mutation Database (HGMD), and Clinvar Databases. Interpretation of the variants followed the recommended standards of the American College of Medical Genetics and Genomics [7]. Finally, Sanger sequencing with target primers was carried out to confirm the variants.

## Results

### Clinical features

**Fig. 1:**
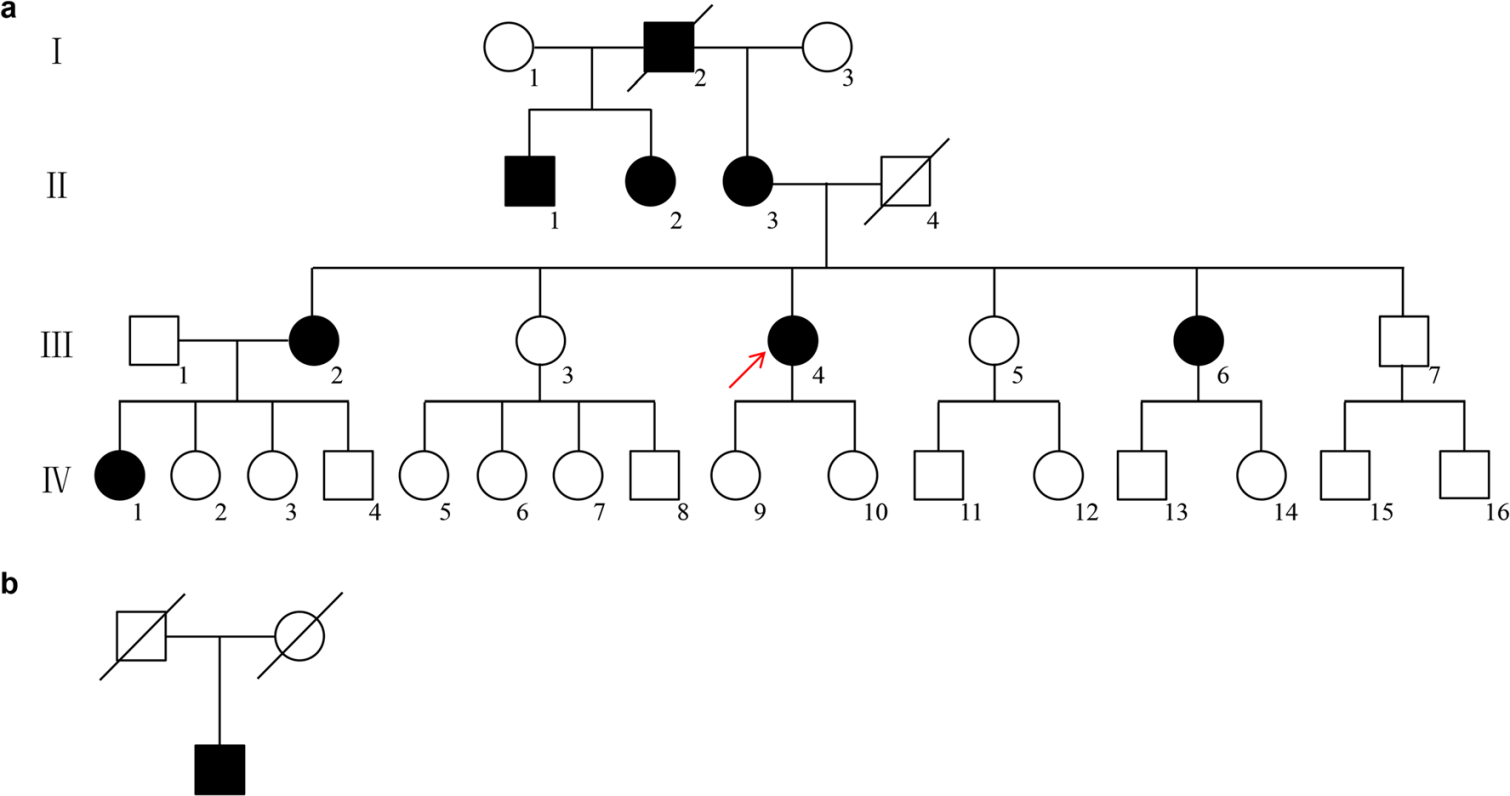
Pedigree of the SCA5 family and the apparently sporadic patient. **a** Pedigree of a Chinese SCA5 family with autosomal-dominant inheritance. The proband and her mother presented with walking instability and progressively getting worse. **b** The sporadic patient suffered fromslurred speech, walking instability, and drinking water choking cough

**Fig. 2:**
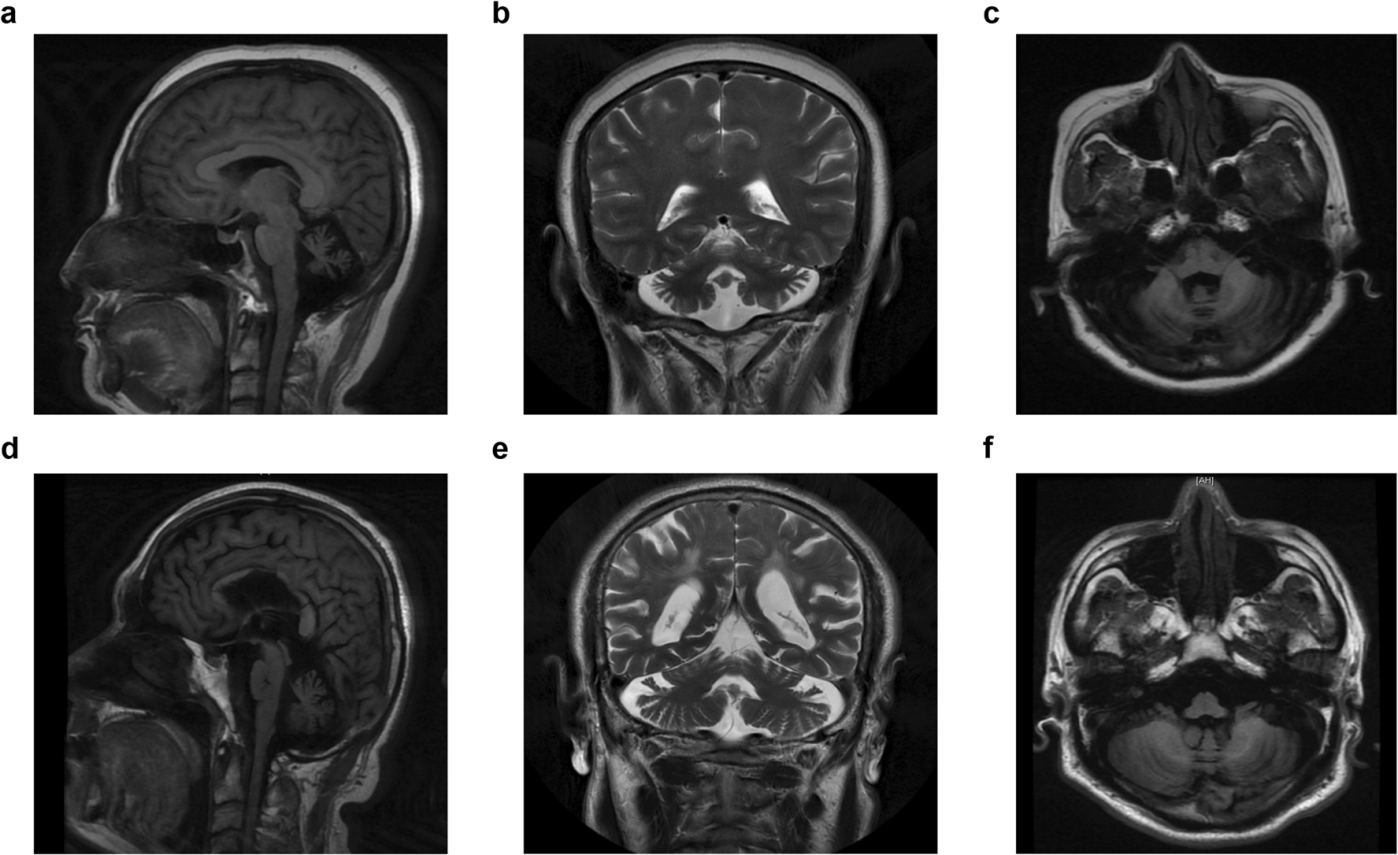
Brain MRI of patients with SCA5. Sagittal (**a** T1-weighted), coronal (**b** T2-weighted), and axial (**c** T1-weighted) brain MRI of the proband of the family with SCA5. Sagittal (**d** T1-weighted), coronal (**e** T2-weighted), and axial (**f** T1-weighted) brain MRI of the sporadic patient with SCA5. They both displayed moderate cerebellar atrophy

The family pedigree demonstrates an autosomal-dominant pattern of inheritance (Fig. 1a). The proband was a 53-year-old female with ataxias for 5 years, and she suffered from walking on cotton and reacting slowly. Her ataxia symptoms have been getting worse especially in the last 1 year before she was referred to our hospital. She got married at 24 years old and had two healthy daughters. She had an obvious family history of cerebellar ataxia. Her mother was 80 years old and began with ataxia at about age 40, dysarthria at age 75, and drinking water choking cough at age 78. Her elder sister and fifth younger sister showed signs of ataxia at about 50 and 44 years old, respectively (Table 1). Her mother was half-sister to her aunt and uncle. Her physical examination revealed dysarthria and cerebellar ataxia. No gaze-evoked nystagmus and pyramidal signs were observed. Her cognitive function assessment scale Mini-Mental State Examination (MMSE) score was 19 and Montreal Cognitive Assessment (MoCA) score was 7. Laboratory analysis revealed normal levels of vitamin B12, thyroid function, and rheumatism series index. Her brain MRI revealed moderate cerebellar atrophy (Fig. 2a–c). Her spine MRI and electromyography (EMG) showed no obvious abnormality.

**Table 1:** Clinical features of the SCA5 family

	Sex	Age of onset	Symptoms
Proband (III 4)	F	48 years	Cerebellar ataxia
II 1	M	50 years	Cerebellar ataxia, dysarthria
II 2	F	55 years	Cerebellar ataxia
II 3	F	40 years	Cerebellar ataxia, dysarthria drinking water choking cough
III 2	F	50 years	Cerebellar ataxia, dysarthria
III 6	F	44 years	Cerebellar ataxia
IV 1	F	32 years	Cerebellar ataxia

The apparently sporadic individual was a 64-year-old male with slurred speech for 10 years and ataxia and drinking water choking cough for more than 4 years (Fig. 1b). His cerebellar ataxia symptoms have been getting worse especially in the last week. He stood unsteadily and was hard to turn around. He visited our hospital last year with partial communication disorders. He had hypertension for 10 years. It was difficult to trace back his family history because his parents passed away. He got married at 30 years old and had a healthy daughter. Physical examination revealed gaze-evoked nystagmus, cerebellar ataxia, and dysarthria. No pyramidal signs were observed. His cognitive function assessment scale MMSE score was 25 and MoCA score was 14. Laboratory analysis revealed normal levels of organic acids in the urine. Brain MRI revealed moderate cerebellar atrophy (Fig. 2d–f).

### Mutation analysis

**Fig. 3:**
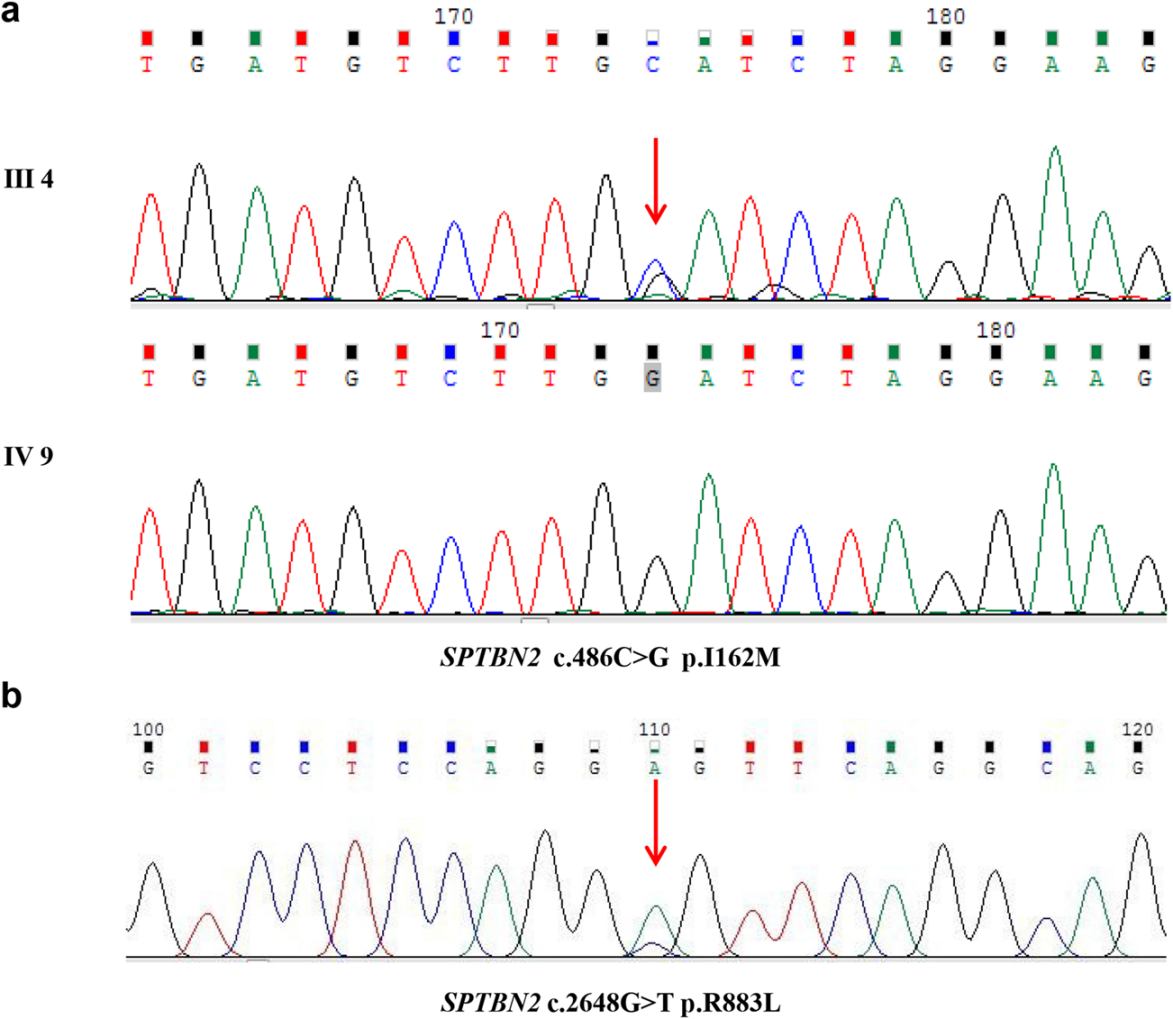
Sanger sequencing of *SPTBN2* mutations identified in the research. **a** Sanger sequencing results of the missense mutation (c.486C>G) in the proband (III 4) and the wide-type sequences (IV 9) in unaffected control. **b** Sanger sequencing results of the missense mutation (c.2648G>T) in sporadic patient

**Fig. 4:**
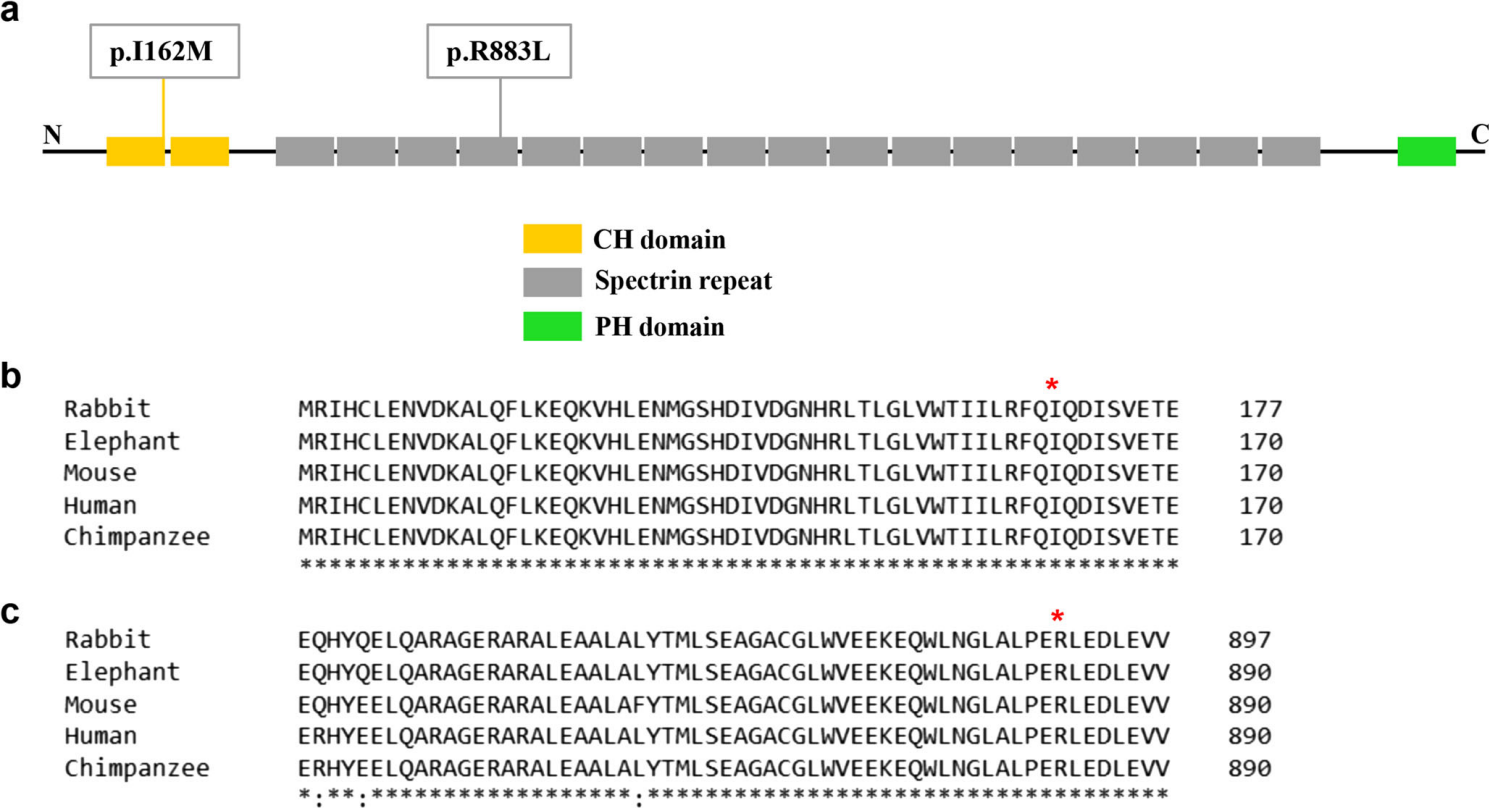
Evolutionary conservation of the identified mutations in *SPTBN2*. **a** Mutations in this work indicated in the schematic representation of β-III spectrin protein. **b** Evolutionary conservation of the mutation p.I162M. **c** Evolutionary conservation of the mutation p.R883L

We verified two novel heterozygous variants in *SPTBN2* by targeted next-generation sequencing and capillary electrophoresis in the genomic DNA of the proband and the sporadic patient. Seven hundred thirty mutations and CAG repeat mutations (data not shown) associated with SCAs were analyzed. A novel missense variant (c.486C>G p.I162M) of *SPTBN2* was found in the proband (Fig. 3a). Sanger sequencing was subsequently performed in the genomic DNA of all family members. We found that the heterozygous variant co-segregated with phenotype of this family. Moreover, the variant was not observed in 150 unrelated healthy control and databases of normal sequence variations (1000 Genome Project and Single-Nucleotide Polymorphism Database). The identified variant was located in the CH domain of β-III spectrin protein (Fig. 4a). The region is highly conserved, with the Ile162 residue found in all five human β-III spectrin proteins as well as in mouse, rabbit, elephant, and chimpanzee (Fig. 4b). We conducted bioinformatic analysis using Polyphen-2 and Mutation Taster software, which predicted that the variants were probably damaging and disease-causing, respectively. Therefore, the discovered variant in *SPTBN2* was likely to be pathogenic.

Targeted next-generation sequencing and capillary electrophoresis were performed to identify the causative gene of the sporadic patient. A heterozygous variant (c.2648G>T p.R883L) of *SPTBN2* was discovered (Fig. 3b). The highest population minor allele frequency of the variant was < 0.005. However, the segregation analysis failed to be performed because his parents were not alive. The variant was located in the spectrin repeat domain and conserved in five species (Fig. 4a, c). The variant was predicted probably damaging and disease-causing by Polyphen-2 and Mutation Taster.

## Discussion

SCA5 is a rare and pure cerebellar ataxia, which was characterized by uncoordinated gait, limb and eye movements, slurred speech, and dysarthria [5]. Onset of SCA5 is in the 3rd or 4th decade, ranging from infancy to 68 years. In this study, we verified two novel heterozygous variants in *SPTBN2*, c.486C>G p.I162M in a pedigree and c.2648G>T p.R883L in the sporadic patient, both with cerebellar ataxias, dysarthria, and cognitive dysfunction by targeted next-generation sequencing. The proband of the pedigree and her mother presented with walking instability and progressively getting worse. Her mother also developed glossolalia and drinking water choking cough. The sporadic patient suffered from slurred speech, walking instability, and drinking water choking cough. MRI examination of the proband and sporadic patient both displayed moderate cerebellar atrophy.

In 1994, Ranum and colleagues mapped the causative gene of SCA5 to the centromeric region of chromosome 11 in a single family descending from the grandparents of President Abraham Lincoln [3]. The SCA5 family had earlier ages of onset in progressive generations. Juvenile onset patients present with evidence of cerebellar and pyramidal trace dysfunction. The second SCA5 family originating from France was described with a slowed progressive cerebellar syndrome. In addition, brisk reflexes, nystagmus, facial myokymia, and decreased vibration sense were displayed in partial patients [8]. Burk et al. reported the third family with autosomal-dominant cerebellar ataxia tightly linked to SCA5 locus from Germany [9]. The patients showed a purely cerebellar syndrome with a downbeat nystagmus occurring prior to the development of other features. Ikeda et al. have discovered that an in-frame variant (c.1592_1630del39; p.E532_M544del) in *SPTBN2* was pathogenic for the 11-generation American kindred [5].

To date, 20 variants of *SPTBN2* have been reported for SCA5: 16 missense variants (p.L626P, p.R480W [10–12], p.R437Q [1, 13], p.R437W [1], p.R437G [14], p.M436T, p.L253P [9], p.I157T, p.T472M [15], p.F160C [1], p.T62I [1], p.R351P, p.H278R [16], p.R721S [17], p.T820M [17], and p.T27I [18]), two three-nucleotide in-frame deletion (c.2608_2610delGAG; p.E870del [19] and c.1276_1278delCTG; p.L426del), and two larger in-frame deletion (c.1886_1900del15; p.L629_R634del [8] and c.1592_1630del39; p.E532_M544del [5]). The variant (p.R480W) was found in three unrelated families. The patients suffered from ataxias with early-onset age and showed global developmental delay. Ellen et al. identified a p.T472M substitution in a late-onset SCA5 family. The proband of the family developed progressive gait ataxia in her early 50s [15]. A heterozygous three-nucleotide in-frame deletion mutation (c.2608_2610delGAG; p.E870del) was detected in the first Japanese family [19]. Five patients in the family presented limb ataxia and dysarthria with late-onset age. Three different variants on the arginine at 437 of spectrin protein including p.R437G, p.R437Q, and p.R437W were reported in four pedigrees. The probands all displayed ataxia, dysarthria, and developmental delay with early-onset age. The phenotypes of probands in previous SCA5 families are shown in Table 2.

**Table 2:** The phenotypes of probands in SCA5 families

Patient	Sex, Age	SPTBN2 variants	Protein	Domain	Age of onset	First symptom	Ataxia	Ocular anomalies	Dysarthria	Pyramidal signs	Tremor	Facial myokymia	Cognitive function	Developmental delay	Additional findings
Current case 1	Female, 53	c.486C>G	p.I162M	ABD	48 years	Ataxic gait	**+**	**−**	**+**	**−**	**−**		MMSE 19 MoCA 7		
Current case 2	Male, 64	c.2648G>T	p.R883L	SPEC (6/17)	54 years	Dysarthria	**+**	**−**	**+**	**−**	**−**		MMSE 25 MoCA 14		Dysphagia
1	Male, 1	c.812C>T [17]	p.T27I	ABD	6 months	Ataxic gait	**+**	−	**+**	Brisk reflexes	−			**+**	Dystonia
2	Female, 2	c.1310G>A [12]	p.R437Q	SPEC (2/17)	3 months	Head nodding	**+**	**+** (Nystagmus)	**+**	Brisk reflexes		−		**+** (Global developmental delay )	Facial hypotonia
3	Female, 6	c.1309C<G [13]	p.R437G	SPEC (2/17)	Infancy	Delayed motor development and hypotonia	**+**	**+** (Gaze-evoked nystagmus)	**+**	−	**+** (Intention tremor)			**+** (Mild intellectual disability; delayed motor development)	
4	Female, 18	c.185C>T [1]	p.T62I	ABD	8 months	Psychomotor delay; microcephaly	**+**	**+** (Horizontal and vertical nystagmus)	**+**	−	−	−		**+** (Cognitive delay)	Mild bradykinesia
5	Male, 5	c.479T>G [1]	p.F160C	ABD	5 months	Psychomotor delay; strabismus	Not acquired	**+** (Strabismus)	**+**	−	−	−		**+** (Cognitive delay)	
6	Male, 18	c.1310G>A [1]	p.R437Q	SPEC (2/17)	5 months	Hypotonia	**+**	**+** (Horizontal nystagmus)	**+**	Brisk reflexes	**+** (Intention tremor)	−		**+** (Cognitive delay)	
7	Female, 8	c.1309C>T [1]	p.R437W	SPEC (2/17)	10 months	Psychomotor delay	**+**	−	**+**	−	−	−		**+** (Cognitive delay)	
8	Female, 2	c.1438C>T [11]	p.R480W	SPEC (2/17)	Congenital	Generalized hypotonia; alternating esotropia	**+**	**+** (Alternating esotropia)	**+**					**+** (Global developmental delay )	
9	Female, 19	c. 833A>G [15]	p.H278R		11 years	Gait ataxia	**+**	**+** (Downbeat nystagmus)							
10	Female, 5	c.1438C>T [10]	p.R480W	SPEC (2/17)	A few weeks	Head nodding; unsteady arm movements	**+**	**+**		−				**+** (Moderate intellectual disability)	
11	Male, 57	c.2608_2610delGAG [18]	p.E870del	SPEC (6/17)	51 years	Poor coordination	**+**	−	**+**	Brisk reflexes			−		
12	Female, 67	c.1415C>T [14]	p.T472M	SPEC (2/17)	50s	Gait ataxia	**+**								
13	Female, 12	c.1438C>T [9]	p.R480W	SPEC (2/17)	12 months	Hypotonic with poor head control	**+**	**+**	**+**	Brisk reflexes	**+** (Intention tremor)	**+**		**+** (Global developmental delay )	
14	Germany pedigree	c.758T>C [8]	p.L253P	ABD	15–50 years	Ataxic gait (10/15)	**+** (14/15)	**+** (Gaze-evoked nystagmus) (13/15)	**+** (13/15)	−	Intention tremor(5/15); Rest tremor(2/15)	−	−		Decreased vibration sense; Reduced tendon reflexes (1/15)
15	Male, 64 (French pedigree )	c.1886_1900del15 [7]	p.L629_R634del	SPEC (3/17)	38 years	Ataxic gait	**+**	**+** (Horizontal nystagmus and gaze)	−	Brisk reflexes	−	**+**	−		Decreased vibration sense
16	American pedigree	c.1592_1630del39 [4]	p.E532_M544del	SPEC (3/17)	Earlier ages of onset in progressive generations; Juvenile onset patients present with evidence of cerebellar and pyramidal trace dysfunction										

SCA5 is rare autosomal-dominant ataxias, especially in Chinese people. Only one of the above variants was reported in a 19-year-old Chinese girl presenting with progressive unsteady gait while running from the age of 11 years. Here, we identified two novel variants of *SPTBN2* responsible for SCA5 in a Chinese family and a 64-year-old male with onset at the fifth decade. It may help to develop genetic counseling and investigate targeted pharmaceutical interventions.

*SPTBN2* encodes β-III spectrin protein, including CH domains (the actin/ARP1 binding site), 17 spectrin repeats involved in the formation of the heterotetrameric α-β-spectrin complex, and PH domains (phosphatidylinositol lipids binding site) [20]. β-III spectrin was primarily expressed in Purkinje cell bodies, dendrites, and axons of the cerebellum. Loss of β-spectrin resulted in marked Purkinje cell loss, dendritic atrophy, and significant thinning of the molecular layer in SCA5 [5]. The mechanism may be decreased sodium currents and deficits in glutamatergic neurotransmission [21–23]. The first variant (c.486C>G p.I162M) in our study was located at CH domain affecting a highly conserved residue. Interestingly, a 5-year-old child with early-onset psychomotor delay and strabismus carrying a different de novo variant but affecting the nearby amino acid (c.479T>C, p.F160C) has been recently reported [1]. Structural analysis revealed that it caused the loss of the hydrophobic interactions with Trp66 and Leu98, modifying the interface of the CH-1 domain with actin. It was predicted that p.I162M may also have a role in regulating the CH-1 domain binding with actin. The second variant in our study (c.2648G>T p.R883L) was located in the 6th spectrin repeats domain which participated in the formation of the heterotetrameric α-β-spectrin complex. The two variants were predicted probably damaging and disease-causing by structure analysis. Functional studies should be urgently needed.

## Conclusion

In this study, we identified two novel heterozygous variants of *SPTBN2* resulting in severe ataxia which further delineated the correlation between the genotype and phenotype of SCA5, and pathogenesis of variants in *SPTBN2* should be further researched.

## Data Availability

The data that support the findings of this study are available from the corresponding author upon reasonable request.
